# Deciphering the Concept of Solubility by Strategically Using the Counterion Effect in Charged Molecules

**DOI:** 10.1021/acs.jchemed.4c00057

**Published:** 2024-07-25

**Authors:** Roberto J. Brea, Armand Hernández, Alejandro Criado, Jesús Mosquera

**Affiliations:** Universidade da Coruña, CICA—Centro Interdisciplinar de Química e Bioloxía, Rúa as Carballeiras, 15071 A Coruña, Spain

**Keywords:** Undergraduate, Experimental teaching, Hands-On Learning, Solubility

## Abstract

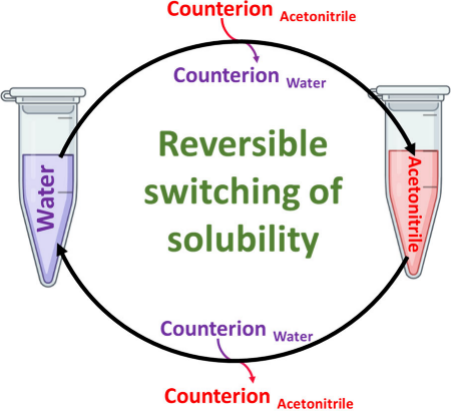

Solubility is an essential concept in chemistry that describes the ability of a substance to dissolve in a particular solvent. Despite its importance in many fields of science, understanding the basic principles of solubility is challenging for many undergraduate students. Notably, students often encounter difficulties in comprehending the role of counterions when dealing with charged molecules. Here, we bring the opportunity to assimilate the key concepts of solubility regarding the role of counterions by developing a straightforward, cheap, and visually appealing experiment that focuses on the strategic use of counterions to control solubility. A student questionnaire delivered encouraging results with most of students giving positive feedback in both interest and training their hands-on skills. Hence, our experiment offers a proficient understanding of the solubility concept, thus preparing undergraduate students for advanced courses in the various subject areas of chemistry.

## Introduction

Chemical reactions can occur in a variety of environments, with solutions being the most common one.^1,2^ In a solution, one or more substances (solutes) are conveniently dissolved in a solvent to create a homogeneous mixture. Solubility is quantitatively expressed as the concentration of solute in a saturated solution at a specific temperature.^3,4^ This relevant property depends on various factors, including the nature of the solute and solvent, temperature, pressure, and the presence of other solutes.^5^ The solubility concept is essential in many fields, such as synthesis, medicine, and industrial processes. For instance, in pharmaceuticals, the solubility of a drug can affect its bioavailability and biodistribution,^6,7^ while in industrial chemistry, it can control the efficiency of chemical processes, particularly in the context of homogeneous catalysis.^8^

The solubilization process entails an equilibrium of the energy associated with three intermolecular force^9^ interactions: solvent with solvent, solvent with solute, and solute with solute.^10^ Solubility relies on the released energy from the interaction between solute and solvent molecules, thus compensating for the other two endothermic processes (i.e., separating solvent molecules from each other and separating solute molecules from each other). Such compensation is typically achieved when a polar solvent interacts with a polar or ionic solute or when a nonpolar solute encounters a nonpolar solvent. For this reason, water is a good solvent for salts, sugars, and similar compounds and mixes in all proportions with alcohol. Conversely, mineral oils dissolve nonpolar substances that are normally insoluble in water.^11^ To simplify the prediction of solubility, the rule of thumb of “Like dissolves like” is often imparted to students.^12,13^ However, caution is warranted when applying this rule to charged molecules, which represent the primary type of molecules involved in biological processes, owing to the influence of the counterion effect.

Interestingly, we have observed that many students tend to focus solely on one part of the structure (either a cation or an anion), particularly when dealing with organic species, while overlooking the role of counterions. However, counterions play a crucial role in determining the solubility of these charged molecules for several reasons. First, counterions influence lattice energy, which is the interaction energy between solute molecules in the solid phase. Second, the interaction between counterions and the solvent can be robust enough to compensate for the low solubility of their complementary ions.^14^ Exemplary manifestations of this phenomenon are apparent in metallic salts, wherein the solubility in water undergoes notable variations based on the nature of the anion. For instance, at 25 °C, silver nitrate exhibits high solubility (2.56 kg/L) in water, whereas silver chloride attains only a concentration of 2 mg/L.^15^

Consequently, the counterion effect emerges as a versatile and powerful tool that can be harnessed to exert control over the solubility of charged molecules. This has far-reaching implications across numerous fields, offering a means to regulate drug solubility,^16^ perform purification purposes, and even enhance the efficacy of various processes such as catalysis.^17^ For instance, a notable application is in reversed-phase chromatography, where trifluoroacetic acid is commonly used as an ion-pairing reagent to increase the hydrophobicity of peptides, thereby improving their retention in the stationary phase.^18^ Furthermore, employing counterions with varying degrees of hydrophilicity as stimuli has shown the capability to reversibly transfer molecular cages between mutually immiscible liquid phases.^19^ This phase transfer phenomenon can be utilized for purifying molecules encapsulated within cages.^20^ Counterions also affect dramatically to the solubility and function of proteins, e.g., Hofmeister effect.^21^ In essence, the influence of counterions extends across a wide array of applications in both chemistry and biochemistry.

Nevertheless, despite its ubiquitous presence and relevance in chemical reactions and processes, the solubility concept is not fully developed or explored in the context of teaching and learning. For instance, to the best of our knowledge, no lab experimental approaches that deal with the counterion effect have been published so far, despite their relevancy. Therefore, innovative active hands-on learning strategies are crucial for improving understanding of this concept. The primary objective of this work was to give students the opportunity to elucidate that solubility is a multifaceted phenomenon extended beyond the simplistic “Like dissolves like” rule.^13^ Specifically, we introduced a cost-effective, visually compelling, and easily understandable experiment to clearly decipher the key concepts of solubility. Moreover, this experiment was designed to facilitate a more profound comprehension of the role of counterions in governing the solubility of their complementary charged molecules. It is also important to recognize that students often perceive solubility as a binary classification of soluble versus insoluble. However, solubility operates along a continuum, with solvents capable of dissolving molecules to varying degrees, ranging from molar concentrations to nanomolar levels.^22^

## Learning Objectives

The experiment outlined in this study was an exceptional opportunity to enrich students’ comprehension of molecular solubility and its manipulation through the strategic use of counterions. The experiment relies upon and reinforces other concepts, including noncovalent interactions, coordination chemistry, and the application of Le Chatelier’s principle.

The synthesis of the coordination complex tris(bipyridine)iron(II) sulfate served as a tangible illustration of coordination chemistry. Beyond this, students understood that not all charged molecules necessarily possess high solubility in water and gained insights into the presence of anions with low hydrophilicity. The application of Le Chatelier’s principle to comprehending the equilibrium shift during the anion exchange process and the subsequent precipitation reaction is a pivotal concept in chemical equilibrium. Thus, the experiment provided a holistic approach to understanding molecular solubility and fundamental principles in chemistry.

## Materials and Methods

### Reagents

2,2′-Bipyridine (CAS: 366-18-7, Purity: 97%) and iron(II) sulfate heptahydrate (CAS: 7782-63-0, Purity: 99%) were purchased form BLDpharm. Tetra-*n*-butylammonium sulfate solution (CAS: 2472-88-0, Concentration: 50% in water) was purchased from ThermoFisher. Potassium hexafluorophosphate (CAS: 17084-13-8, Purity: 99%) was purchased from Sigma-Aldrich. Acetonitrile (HPLC Gradient Grade) was purchased from Thermo Scientific Chemicals. It is important to note that acetonitrile has the capacity to absorb significant amounts of water from the environment if left exposed to the air, resulting in inefficient precipitations. Milli-Q water was used as aqueous solvent.

### Instruments

A desktop laboratory centrifuge is required to perform this experiment. Ideally, the centrifuge should attain 5,000 rpm; however, lower revolutions can be compensated for by extending the centrifugation time. Students must be provided with a brief instructional guide on operating the centrifuge. Specifically, they should be made aware that running an unbalanced centrifuge could result in considerable damage and pose a risk of injury to the operator and other laboratory personnel and objects. Hence, it is imperative to employ centrifuge tube pairs with closely matched weights, ensuring that the difference in mass between them does not exceed 50 mg.

## Results and Discussion

This experiment was conducted by students enrolled in the third-year course on Supramolecular Chemistry, as part of the Nanoscience and Nanotechnology degree program at Universidade da Coruña. They were carried out during the spring semesters of both 2023 and 2024. An average of 30 students participated in these exercises annually. It is worth noting that this experiment is also suitable for second-year students enrolled in Chemistry degree programs, especially those emphasizing topics in general organic and inorganic chemistry. During the exercise, the students were divided into groups of two participants. The experiment extended for approximately 2 h. Previously, all students received a half-hour introduction to solubility (see “Notes for the Instructor” in Supporting Information for more details). During the experiment, students were required to write a laboratory notebook, which must be submitted upon completing the experiment. A guide for creating the laboratory notebook is provided in the Supporting Information. The evaluation of this notebook was conducted according to the rubric provided in the Supporting Information (Table S1). Additionally, the questions provided in the Supporting Information were intended to assess students’ mastery of the concepts.

To conveniently prepare the students for the planned experiment, the instructor initially introduced the most commonly used solvents in chemistry, highlighting their properties and applications. The concept of “Like dissolves like”^13^ was also discussed, taking advantage of solvent miscibility, and then providing examples of different types of molecules and their solubility in specific solvents. It was important to remind students of the various types of noncovalent interactions that are responsible for solubilization, such as hydrogen bonding, ionic interactions, and van der Waals forces. A clear understanding of these concepts is critical for the success of our experiment and for future endeavors in the field of chemistry.

Among these interactions, hydrogen bonding often presents significant challenges for students. Defined by the IUPAC as an attractive interaction, hydrogen bonding involves a hydrogen atom bound to a molecule or molecular fragment X–H, where X is more electronegative than hydrogen. This interaction occurs with an atom or group of atoms in the same or a different molecule, where evidence of bond formation is observed. The driving forces behind hydrogen bonding include electrostatic interactions and charge transfer between the donor and acceptor, resulting in partial covalent bond formation between hydrogen and the acceptor atom or group Y.^23^

Another topic that should be introduced by the instructor, and can be confusing for students, is the difference between molecular polarity and solvent polarity. A polar molecule is defined as one containing polar bonds where the sum of all the bond dipole moments is not zero. Thus, molecular polarity is related to the symmetry of the molecule. For example, acetonitrile, with a dipole moment of 3.44 D, is more polar than water, which has a dipole moment of 1.87 D. Surprisingly, very hydrophilic anions like sulfate or nitrate are considered apolar under this definition.

In contrast, solvent polarity is a bulk property related to the overall solvation capability for solutes, determined by all possible intermolecular interactions with solute molecules. Generally, the static dielectric constant is used to quantify solvent polarity, as a higher dielectric constant typically indicates a better ability to dissolve polar and ionic compounds.^24^ Under this definition, water is considered a protic, highly polar solvent with a dielectric constant of 78, while acetonitrile is an aprotic medium-polarity solvent with a dielectric constant of 35.

To understand the marked difference in the solubility of the tris(bipyridine)iron(II) complex depending on the counterion, it is crucial to delve into the contrasting hydrophilicity of the hexafluorophosphate and sulfate anions. Sulfate is widely acknowledged as one of nature’s most hydrophilic anions, largely attributed to its −2 net charge, small size and the presence of four oxygen atoms capable of serving as hydrogen bonding acceptors. This characteristic enables sulfate to engage in numerous hydrogen bonds with water molecules, thereby enhancing its solubility. Conversely, hexafluorophosphate is recognized as one of the less hydrophilic anions for the following reasons, (i) it has a single charge; (ii) its negative charge is very diffuse, as it is shared by the six highly electronegative fluorine atoms surrounding the central phosphorus atom, leading to weak hydrogen bonds and ion-dipole interactions with water;^25^ and (iii) it is a relatively large anion that requires more space in solution, resulting in a greater free-energy penalty for hydration.^26^ Consequently, acetonitrile is well-suited for dissolving charged molecules with low hydrophilicity, such as hexafluorophosphate anions. This distinction explains why the solubility of the tris(bipyridine)iron(II) complex is maximized in water for the sulfate salt and in acetonitrile for the hexafluorophosphate salt.

This experiment comprised several steps:

### 1 Synthesis of Tris(Bipyridine)Iron(II) Sulfate.

**Figure 1:**
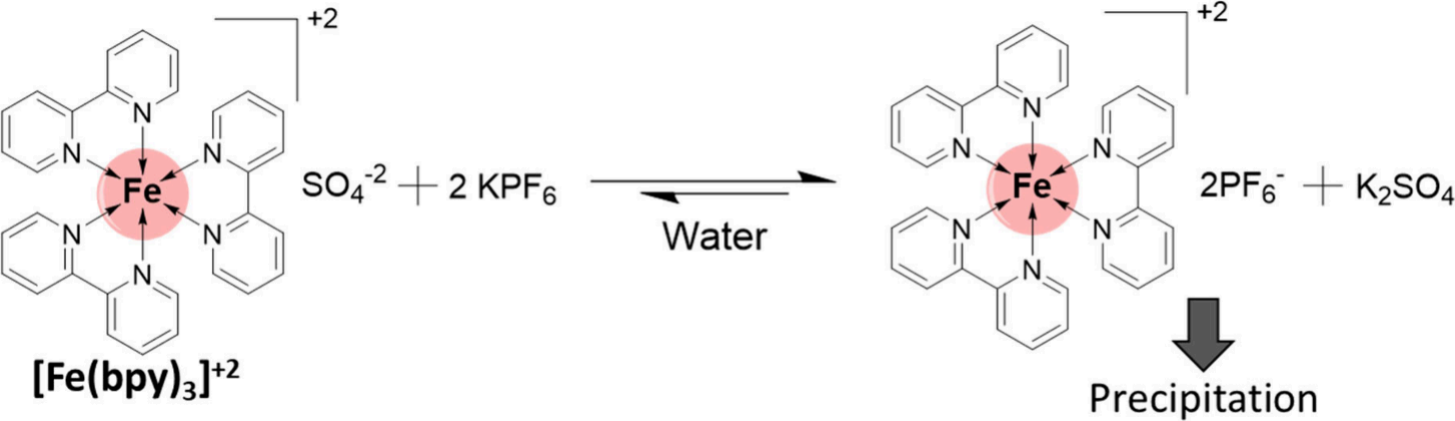
Schematic representation of the equilibrium involved in the precipitation of tris(bipyridine)iron(II) complex by the addition of potassium hexafluorophosphate.

**Figure 2:**
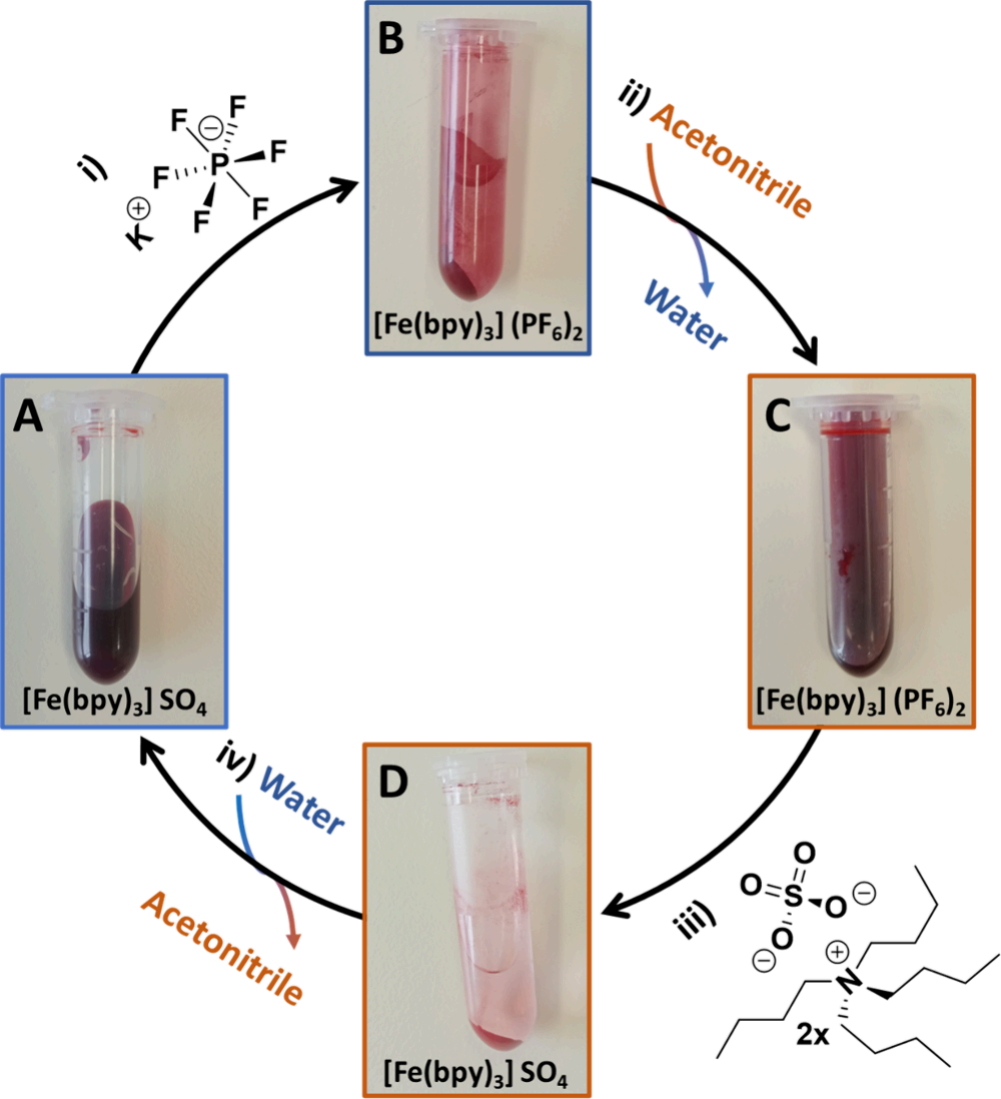
Schematic representation of the procedure to follow in order to reversibly control the solubility of the complex ion tris(bipyridine)iron(II), [Fe(bpy)_3_]^2+^.

In this coordination complex, iron forms an octahedral arrangement with three bipyridine ligands (Figure 1),^27^ resulting in an intense orange color due to metal-to-ligand charge transfer. This complex was selected for its striking color, which captured the students’ attention and facilitated the observation of the process. Moreover, it can be easily synthesized using a simplified one-step procedure adapted from a previously reported method.^7^ Each student placed 2,2′-bipyridine (7 mg, 43 μmol) and iron(II) sulfate heptahydrate (4 mg, 14 μmol) in a 2 mL microcentrifuge tube. Then, they added 1 mL of distilled water, and hand-shook the mixture for 30 s. The resulting solution immediately turned from colorless to dark orange, indicating the corresponding formation of the complex ion tris(bipyridine)iron(II) (Figure 2A). The complex readily dissolves in water when paired with the hydrophilic sulfate counterion, but it exhibits very low solubility in acetonitrile (20 μM, Table 1). The hydrogen bonds established between sulfate ions and water molecules are responsible for promoting solubility.

**Table 1:** Experimentally Determined Solubilities of Tris(Bipyridine)Iron(II) Salts in Water and Acetonitrile at Room Temperature Obtained by UV/Vis Spectroscopy^28^

	Water solubility	Acetonitrile solubility
[Fe(bpy)_3_]SO_4_	0.4 M	20 μM
[Fe(bpy)_3_](PF_6_)_2_	260 μM	0.5 M

### 2 Transforming Tris(Bipyridine)Iron(II) Sulfate into a Salt with Low Solubility in Water [Figure 2B, Step (i)]

After synthesizing the iron complex, the next step was to convert it into a salt with low solubility in water by exchanging highly hydrophilic sulfate counterion with the less hydrophilic hexafluorophosphate. Students added an excess of potassium hexafluorophosphate (15 mg dissolved in 150 μL of water, 81 μmol) to the previous tube (containing the iron complex aqueous solution). Then, they shook the microtube for a few seconds, resulting in the formation of a large amount of orange solid. To isolate the solid complex, students centrifuged the mixture for 1 min at 5,000 rpm. After centrifugation, the aqueous supernatant liquid remained colorless, indicating the complete precipitation of the complex.

This phenomenon can be explained by Le Chatelier’s principle, as shown in Figure 1. The precipitation of tris(bipyridine)iron(II) hexafluorophosphate reduced its concentration in the solution, causing the equilibrium to continuously shift toward further formation and precipitation. Ultimately, the tris(bipyridine)iron(II) was precipitated as the PF_6_ salt, while potassium sulfate remained in solution, along with the excess of potassium hexafluorophosphate. It is noteworthy that potassium hexafluorophosphate is highly water-soluble (0.45 M)^29^ due to the hydrophilic nature of the potassium cation, compensating for the low hydrophilicity of the hexafluorophosphate anion. In contrast, the solubility in water of the tris(bipyridine)iron(II) hexafluorophosphate salt is 260 μM and the sulfate salt is 0.4 M (Table 1).

### 3 Dissolving the Tris(Bipyridine)Iron(II) Hexafluorophosphate in Acetonitrile [Figure 2C, Step (ii)]

After centrifugation of the tris(bipyridine)iron(II) complex solid, students carefully removed the water from the tube using a Pasteur pipet. Subsequently, they added acetonitrile (1 mL), resulting in the immediate solubilization of the solid material. Due to the characteristics of hexafluorophosphate as explained above, this anion demonstrates an ideal interaction with medium-polarity solvents such as acetonitrile, achieving a solubility of 0.5 M.

### 4 Transforming Tris(Bipyridine)Iron(II) Hexafluorophosphate into a Salt with Low Solubility in Acetonitrile [Figure 2D, Step (iii)]

The previous steps of the protocol allowed for the synthesis of an iron complex characterized by low solubility in water but high solubility in acetonitrile. To reverse this situation and reform the water-soluble version, the students added tetra-*n*-butylammonium sulfate (50 μL of a 50% w/w aqueous solution, 43 μmol), which immediately induced the precipitation of the complex as a sulfate salt.The former addition induced the formation of two distinct salts. The first is tetra-*n*-butylammonium hexafluorophosphate, which exhibits high solubility in acetonitrile due to the medium hydrophilicity of both ions, allowing it to remain dissolved in this solvent. The second salt comprises the most hydrophilic ions, the cationic complex, and the anionic sulfate. Unlike the first salt, these ions have net charges of +2 and −2, respectively. Additionally, since acetonitrile is an aprotic solvent, the sulfate ion cannot form hydrogen bonds with it, resulting in the low solubility of the complex. Consequently, this salt immediately precipitates (Figure 2, Eppendorf D). After centrifugation (1 min at 5,000 rpm), all the orange solid stayed at the bottom of the tube, and the acetonitrile became colorless [Figure 2, step (iii)]. The same reasoning applied in the previous step can be used to explain the precipitation of the iron complex due to the formation of the sulfate salt, which exhibited low solubility in acetonitrile.

### 5 Dissolving the Tris(Bipyridine)Iron(II) Sulfate in Water [Figure 2A, Step (iv)]

In the last stage of the experiment [Figure 2, step (iv)], the acetonitrile was cautiously removed from the vial using a Pasteur pipet, and the addition of water led to the instant solubilization of the material (Figure 2, Eppendorf A). It is worth noting that the cycle shown in Figure 2 could be repeated multiple times, thanks to the almost quantitative yield achieved in each step. This aspect provided a great opportunity for students to observe the reversible nature of solubility through the controlled addition or removal of counterions with quite distinct hydrophilicity. Although the decrease in water solubility of the corresponding complexes is mainly a consequence of the incorporation of ions with low hydrophilicity (i.e., PF_6_^–^ anions), we should also consider that ions of similar size usually make more stable lattices, thus reducing the solubilities of the obtained species. Moreover, the solvation energy of the ion is also important in determining solubility.^31^ Low solvation energies (usually associated with big cations) tend to decrease the solubility of the ionic substances.

## Hazards and Waste Disposal

For safety reasons, it was imperative that students adhered to proper safety precautions while conducting this experiment. This included wearing all required personal protective equipment (PPE), such as a lab coat, safety goggles, and gloves. The chemicals used in the proposed experiment, such as 2,2′-bipyridine, iron(II) sulfate heptahydrate, potassium hexafluorophosphate, and tetra-*n*-butylammonium sulfate, are toxic if swallowed or in contact with skin.

Acetonitrile is a highly flammable liquid that can be easily ignited by heat, sparks, or flames. When heated, it can give off highly toxic hydrogen cyanide fumes. Acetonitrile dissolves easily in water, but it can also react with water, steam, or acids to produce flammable vapors that can form explosive mixtures when exposed to air. Therefore, it was essential to handle acetonitrile with extreme care, and it should only be used in a well-ventilated area with appropriate safety measures in place.

Every student in the class received instructions to discard all used solutions into a designated waste container. All chemical waste and remaining materials were disposed of in strict accordance with Safety Data Sheets (SDSs).

## Feedback

Sixty students in the fourth semester participated in this exercise, with 28 of them responding to the questionnaire (Table 2) using Google Forms. Concerning the first question, 42.9% of the students indicated familiarity with solubility knowledge. However, in response to the second question, 64.3% of these participants acknowledged the experiment’s significant utility in enhancing their understanding of this subject. As for the third question, 92.9% of the students were interested or very interested in the laboratory experiment, likely due to the opportunity to explore into more advanced concepts in supramolecular chemistry (35.7%) or observe intriguing experimental phenomena (28.6%). Indeed, most participants found enjoyment in the experiment, particularly in witnessing the appearance and disappearance of precipitates in the solution. Regarding the fifth question, over 70% of the students reported that this exercise was beneficial or highly beneficial in training their hands-on skills.

**Table 2:** Questionnaire and Feedback

Questions	Options	Results (%)
What knowledge did you have about solubility of charged molecules before the subject “Supramolecular Chemistry”?	Familiar	21.4
	Known	42.9
	General	35.7
	Not at all	0
Does this lab experiment help you to deepen your knowledge of solubility of charged molecules?	Very useful	64.3
	Useful	28.6
	A bit useful	7.1
	No use	0
Are you interested in this laboratory experiment about the effect of the counterion on the solubility of charged molecules?	Very interested	28.6
	Interested	64.3
	Little interested	7.1
	Not interested	0
	No idea	0
You are mostly interested in this laboratory experiment about solubility of charged molecules because...	I have been able to learn more advanced knowledge in the field of supramolecular chemistry.	35.7
	I have been able to observe interesting experimental phenomena	28.6
	I have been able to synthesize useful material	0
	I have been able to develop my scientific thinking	14.3
	Others	0
What do you think of the effect of this exercise on your laboratory skills acquisition for chemical experiments?	Highly beneficial	14.3
	Beneficial	57.1
	Average	14.3
	Bad	7.1
	No idea	7.1

## Conclusions

The experiment described in this manuscript was not only simple and inexpensive but also offered a great opportunity for students to develop their critical thinking and problem-solving skills. By performing this experiment, students gained a better understanding of: (i) the important concept of solubility; (ii) noncovalent interactions; (iii) Le Chatelier’s principle. Furthermore, this experiment provided a hands-on experience for undergraduate students in the lab and helped them become familiar with common laboratory techniques, including centrifugation, the preparation of solutions, and the use of organic solvents. The success of this experiment is reflected in the grades achieved this year by the students, where in two consecutive academic years we have had a 53% of “A” grades (Table S2).

Overall, the planned experiment provided an excellent way for students to learn important chemical concepts and gain practical laboratory experience. In addition, understanding these concepts is essential for the prospective advancement of students across various chemistry disciplines, ranging from chemical engineering to biochemistry. In industrial applications, recognizing the significance of solubility in product purification and crystallization is essential for influencing product quality and yield, particularly in pharmaceuticals and chemicals. Noncovalent interactions are indispensable for material design and surfactant processes across various industries, while acid–base processes contribute to the production of diverse chemicals, fuels, and pharmaceuticals. Le Chatelier’s principle assumes a pivotal role in guiding reaction optimization and ensuring process stability, a critical factor in maintaining operational efficiency in industry. Moreover, in the field of biochemistry, this foundational knowledge is central for comprehending biomolecular behavior, facilitating drug design, and navigating the interdisciplinary landscape with a comprehensive and informed approach.

## Supporting Information Available

Student handout, instructor notes, grading rubric for laboratory notebook, and questions for students (PDF)
