# Supplementary material for: Deciphering the Concept of Solubility by Strategically Using the Counterion Effect in Charged Molecules
Source: J Chem Educ. 2024 Jul 25;101(8):3390–5. doi: 10.1021/acs.jchemed.4c00057 (PMC11331533; doi:10.1021/acs.jchemed.4c00057)
Supplement: Supplementary file 1 — ed4c00057_si_001.pdf [file ed4c00057_si_001.pdf]

SUPPORTING INFORMATION

Deciphering the concept of solubility by strategically using the counterion effect in charged molecules

Roberto J. Brea,[‡,a] Armand Hernández,[‡,a] Alejandro Criado,[*,a] Jesús Mosquera[*,a]

[a] Universidade da Coruña, CICA – Centro Interdisciplinar de Química e Bioloxía, Rúa As Carballeiras, 15071 A Coruña, Spain.

Corresponding author's e-mail address: a.criado@udc.es and j.mosquera1@udc.es

CONTENTS:

Student handout 2

Notes for the instructor...4

Instructions for the laboratory notebook preparation.....................5

Grading rubric for assessment...7

Questions for students...8

Student handout

Exploring the effect of counterions in solubility

Introduction

In a solution, one or more substances, called solutes, are dissolved in a solvent to create a homogeneous mixture. Solubility is defined as the maximum amount of a solute that can be dissolved in a solvent at a specific temperature and pressure. Therefore, this physical property is mainly controlled by intermolecular forces established between the solvent and the solute. Such forces are weak and reversible, being usually called supramolecular interactions. In general, high solubility implies that the forces between the solute and solvent molecules must be stronger than those between the solute molecules. This normally occurs when both the solute and the solvent have similar polarity. Thus, the rule of thumb coined "Like dissolves like", is a simple way to predict solubility.

When working with charged molecules, it is crucial to consider all the salt components since counterions can significantly affect the solubility of molecules. Herein, you will explore the significant effects of counterions in solubility and the limitation of the "Like dissolves like" rule by performing a straightforward experiment.

Instructions

You will need a lab notebook to carefully record steps and collect data from the experiment described below. For each step, include the table of reagents and the reaction scheme.

Experimental procedure.

Controlling the solubility of a complex in different solvents, such as water and acetonitrile, can be achieved by following the corresponding steps:

1. In a 2 mL microcentrifuge tube, add 2,2'-bipyridine (7 mg, 43 μmol) and iron(II) sulphate heptahydrate (4 mg, 14 μmol). Then, add 1 mL of water and shake the tube manually for 30 seconds. Note down the result in your notebook.

2. Add an excess of potassium hexafluorophosphate (15 mg dissolved in 150 μL of water, 81 μmol) to the previous solution. Shake the tube briefly. Then, centrifuge the tube at 5,000 rpm for 1 min. Carefully remove the water from the tube using a Pasteur pipette and subsequently dissolve the resulting orange solid in acetonitrile by hand shaking.

3. Add tetra-*n*-butylammonium sulphate (50 μL of a 50% w/w aqueous solution, 43 μmol) to the previous acetonitrile mixture, and then shake the tube briefly. Then, centrifuge the mixture again at 5,000 rpm for 1 min. Afterwards, carefully remove

the acetonitrile from the tube using a Pasteur pipette and dissolve the resulting orange solid in water by hand shaking.

4. Discuss the results and interpret them.

Notes for the instructor

Timing and lab lecture:

- Time 30 min: short explanation about solubility, supramolecular interactions, acetonitrile and water properties as a solvent, and the "Like dissolves like" rule.
- Time 1.5 h: Students perform the experimental procedure.
- Time 30 min: Discussion of the results of the experiment with the students.

Alternative procedures:

The experiment procedure has been explained in the student handout. Here, we present several alternatives that may be useful and can provide flexibility to the instructor.

- The number of centrifugation revolutions required in the experimental procedure can be reduced by directly increasing the time of each centrifugation step. Step 3 requires special attention since the high viscosity of water could cause the solid adheres to the tube wall, thus making difficult to observe the transparency of the liquid. Slight extension of centrifugation time would be valuable to counteract this issue.
- The solubilization steps in the experimental procedure can be speed up by sonication or using a mixer (i.e., shaker, vortex).
- The potassium hexafluorophosphate can be replaced by sodium hexafluorophosphate.
- If the precipitation in Steps 2 and 4 is not efficient (the supernatant liquid is not colourless), consider adding small additional amounts of potassium hexafluorophosphate or *n*-butylammonium sulfate until the liquid becomes colourless. This will improve the efficiency of the precipitation process.

Instructions for the laboratory notebook preparation

The students will write a lab report to describe the activities related to the solubility of charged molecules by ion exchange. This report will consist of: i) a table of content that will be included in the first page, indicating the page number where each experiment begins, ii) first section about the synthesis of tris(bipyridine)iron(II) sulfate and iii) the second section about the solubility switching based on the ion exchange (see figure 1 in the main manuscript). This third section will be divided into: iiia) ion exchange with potassium hexafluorophosphate and solubilization in acetonitrile (steps 2 and 3), iiib) ion exchange with tetrabutylammonium hexafluorophosphate and solubilization in water (steps 4 and 5). Each experimental section will include the following structure:

(1) Heading: the starting date of the experiment and the title of the practical should be stated.

(2) Introduction: it should include (a) a scheme for each of the three chemical reactions (i.e., formation of the iron complex and the two solubility equilibria) to be performed, (b) a table with the stoichiometric calculations necessary to perform the experiments, (c) the risks associated with the equipment and the chemical compounds (reagents and solvents), as well as the necessary recommendations for their correct handling (H and P phrases), (d) the physical and chemical properties of the compounds that are of interest for the development of the experiment (melting points, boiling points, solubility, acidity or pKa constants, etc.), (e) a graphic description of the setups that are intended to be used in the development of the experiment, (f) a description of the physical and chemical properties of the compounds that are of interest for the development of the experiment, (e) an explanatory diagram of the separation process of the compounds including the name of the operations, the fractions generated and their composition.

When a reaction process is carried out, the scheme must include the structure/chemical formula of the reactants and an indication of the solvent, and the conditions used.

The table with stoichiometric calculations should include 8 columns: (1) name or formula of the compounds, with an indication of their purity, (2) molecular weight (MW, in g/mol) to at least one decimal place, (3) mass (in mg), (4) density of liquids (d, in g/mL), (5) volume (V, in mL), (6) quantity of mol (n, in μmol), (7) equivalents (equiv., the molar ratio to the limiting reagent) and (8) observations, including a few words on the H and P phrases associated with the compound. In the rows of the table corresponding to each compound it will not be necessary to include all the data, only those that are essential should be completed.

As an example, the heading, the chemical reaction for the iron complex formation, the table with the stoichiometric calculations and the risks associated with the chemical compounds used are included below:

Synthesis of tris(bipyridine)iron(II) sulfate

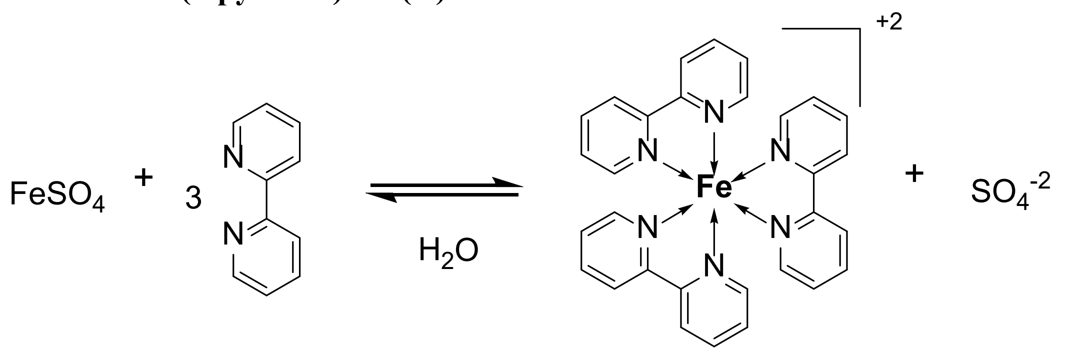

Compound	**MW** (g/mol)	**mass** (mg)	**d** (g/mL)	**V** (mL)	**n** (µmol)	**Equiv.**	**Observations**
$FeSO_4 \cdot 7H_2O$	169,9	4,0			14,0	1	Harmful if swallowed. Causes skin irritation. Causes serious eye irritation
$C_{10}H_8N_2$	156,1	7,0			43,0	3	Acute toxicity, oral and dermal; Causes serious eye irritation; Harmful to aquatic life with long lasting effect.
H_2O				1,0			

(3) Procedures. This section should include a brief, clear and precise description of all the experimental operations carried out in this experiment, which should be written as it is developed. Therefore, it will be written what has actually been done and not what will be done or what is supposed to have been done. It is not a question of reproducing literally the script of the practice, but it is necessary to describe, in a very brief way, the material used, the place of the laboratory where you have worked, the order of addition of the compounds, the observed changes, state of the mixtures (precipitates, dissolution of solids, etc.) or in the appearance (coloration) of the system. As far as possible, such changes should be interpreted, but always in relation to a theoretical model that allows rationalization of the experiment, i.e., in terms of a chemical mechanism.

It should be emphasized that unsuccessful experiments provide information on aspects to be avoided in subsequent experiments and should therefore be described with the same level of detail.

(4) Results and conclusions. The appearance, state of aggregation, and homogeneity of the products should be described. Finally, in the conclusions a critical analysis of the results, sources of error, possible improvements of the procedure or other recommendations for further work can be made.

Grading rubric for assessment

Table S1. Experiment reports assessed students' ability to write the laboratory notebook.

**Laboratory notebook**	**Evaluation / total: 10 pts**
Significant figures and units. 0.1 deduction every time they are not present or incorrect.	
Heading and introduction.<br>Discusses theoretical background of the lab based on the lab objectives.<br>No credit if it is only a summary of the experimental section or rephrasing of the overview section of the protocol.	1.5
Procedure.<br>Gives a summary with key steps of experiment. Uses experimental values where applicable.	1
Results and Discussion.<br>• Representation for the chemical reaction and the two ion exchange equilibria. (1 pt).<br>• Calculation of equivalents of the reagents (1 pt).<br>• Explanation of the solubility of each iron complex in the corresponding media paying attention to the solvent-solute chemical interactions (2 pts).	4
Conclusions.<br>• Answers to the five questions of the lab are included in the text of the conclusion (no question-and-answer format).<br>• Identifies sources of error and proposed improvements.	2
References.<br>Proper use of citations and references. Uses one reference system.	0.5
Overall feeling of report.<br>• Grammar and scientific writing (0.5 pts).	1

• Sections, tables, and chemical representations are organized and clear (0.5 pts).	

Questions for students

Questions related to the experimental procedure:

• Why is $Fe(bpy)_3^{2+}$ so colorful?

Answer: *In transition metal complexes, a change in electron distribution between the metal and a ligand gives rise to charge transfer (CT) bands. In the particular case of* $Fe(bpy)_3^{2+}$*, the intense orange color is a consequence of the metal-to-ligand charge transfer (MLCT) that occurs from Fe(II) to the empty π* orbitals of bpy ligand.*

• Which of the $Fe(bpy)_3^{2+}$ salts used in the experimental procedure are more soluble in ethyl acetate?

Answer: *Ethyl acetate is classified as a polar aprotic solvent, thus being an adequate solvent for dissolving medium-polarity molecules like hexafluorophosphate anions. Taking this into consideration, tris(bipyridine)iron(II) hexafluorophosphate would be the more soluble salt in ethyl acetate.*

• How do you predict the solubility behavior of the $Fe(bpy)_3^{2+}$ chloride salt? Explain your answer.

Answer: *The hydrophilic nature of the chloride anion enables it to engage in numerous hydrogen bonds with water molecules, thereby enhancing its solubility on this polar solvent. Therefore, the* $Fe(bpy)_3^{2+}$ *chloride salt would present a higher solubility in water (polar solvent) and a lower solubility in nonpolar solvents (e.g., hexanes).*

• Is it accurate to state that $Fe(bpy)_3(PF_6)_2$ is completely insoluble in water?

Answer: *It is inaccurate to say that a given compound is "completely insoluble in a particular solvent." In practice, there is almost always some degree of solubility for any compound in any solvent. This solubility can be extremely low, sometimes in the nanomolar range or even less, making the dissolution of the solid imperceptible.*

• Is acetonitrile miscible with water? Is chloroform miscible with water? Is chloroform soluble in water?

Answer: *Miscibility is defined as the ability of two solvents to mix in all proportions, forming a homogeneous solution. If two solvents are miscible, they are also completely soluble in one another irrespective of the order of introduction and ratio.*

Solvents with similar polarities, indicated by similar dielectric constants, are typically miscible. For instance, water, with a dielectric constant of 80, is miscible with acetonitrile at room temperature, which has a

dielectric constant of 37.5. However, water is immiscible with chloroform, an apolar solvent with a dielectric constant of 4.8.

In terms of solubility, chloroform has a relatively high solubility in water (8 g/L at 20°C) compared to other molecules, such as carbon tetrachloride (0.8 g/L at 20°C). Although chloroform is considered an apolar solvent, it is actually a polar molecule (as explained in the manuscript), which enhances its solubility in water. In contrast, carbon tetrachloride, despite having a similar dielectric constant (2.2), is a nonpolar molecule, resulting in its much lower solubility in water.

Table S2. Percentage of qualifications for students that have performed this experiment during the courses 22/23 and 23/24. The total number of students was 60.

Qualification range	Weight / %
10-9	53
<9-8	32
<8-7	8
<7-5	3
<5	3
